# Closed Shop or Collaborative Hub? An Analysis of the Partners' Importance in CANZUK Countries' Research Collaborations

**DOI:** 10.3389/frma.2022.838553

**Published:** 2022-07-26

**Authors:** Ba Xuan Nguyen, Jesse David Dinneen, Markus Luczak-Roesch

**Affiliations:** ^1^School of Information Management, Victoria University of Wellington, Wellington, New Zealand; ^2^Faculty of Business Administration, Posts and Telecommunications Institute of Technology, Ho Chi Minh City, Vietnam; ^3^Berlin School of Library and Information Science, Humboldt-Universität zu Berlin, Berlin, Germany; ^4^Te Punaha Matatini, Aotearoa New Zealand's Centre of Research Excellence for Complex Systems, Auckland, New Zealand

**Keywords:** international research collaboration, measurement, collaboration network, research partner, counting method, CANZUK countries

## Abstract

Collaborative partners are important in international research collaboration. The research collaborations between four CANZUK countries (Canada, Australia, New Zealand and the United Kingdom) are examined to see whether their research connections are different from the research relationships with other countries. This paper measures the affinity index values and analyses the development of research collaborations among CANZUK countries with those between the CANZUK and other countries. The whole counting method and the fractional counting method are applied in this study to compare the differences in the results. The findings show that although the affinity index values of CANZUK countries were decreasing over time, the importance of CANZUK partners to CANZUK countries has likely increased over time at the expense of the other partners' importance. The study also shows the minor differences in results obtained by applying two different counting methods. These differences can be explained by the nature of the counting methods, and the choice to use either one of these two counting methods should be considered in other international research collaboration studies.

## Introduction

International research collaboration (IRC), which refers to scientific collaborations between individuals from different countries, has developed rapidly in recent years (Wagner and Leydesdorff, 2005). Many countries have encouraged policies supporting IRC (Pohl, 2020). The encouragement is because it has been suggested that IRC results in higher scientific impact (Glänzel and Schubert, 2001) and higher productivity (Castillo and Powell, 2020). As the distinctive characteristics of one country may affect the productivity growth of its partners (Baty et al., 2017), the prioritization of countries for collaboration in research is an important strategic decision (Hatakenaka, 2008).

This paper investigates the importance of research collaborations for CANZUK countries: Canada (CAN), Australia (AUS), New Zealand (NZL), and the United Kingdom (GBR).^1^ The countries in this group have traditionally demonstrated economic and political links (Bell and Vucetic, 2019), and have formed a separate research cluster in the IRC network (Davidson Frame and Carpenter, 1979; Schubert and Braun, 1990; Luukkonen et al., 1993; Wagner and Leydesdorff, 2005). However, no prior study has explored the importance of research partners among CANZUK countries.

This paper aims to answer three research questions (RQs):

- RQ1: How has the importance of partners for research collaborations among CANZUK countries developed over time?
- RQ2: How has the importance of partners for research collaborations between CANZUK and other countries developed over time?
- RQ3: How do different methods of counting research collaborations show different results?

RQ1 and RQ2 are answered below by comparing the importance of CANZUK and non-CANZUK countries (i.e., their partners) to research collaborations from 1951 to 2017, for which we use different measures (i.e., counting methods) of importance over bibliographic data. RQ3 is then answered by comparing the nature and results of the counting methods utilized. Finally, we discuss the implications of these answers for interpreting CANZUK research collaboration and for future research selecting among measures of importance.

## Related Work

### Studies About Research Collaboration Networks

Methods from social network analysis have been commonly used to investigate research collaborations across countries. Table 1 below shows these IRC network studies with their data range of IRC publications under survey. The prominent IRC network studies have mainly surveyed the IRC networks in the period 1981–2000. The networks have likely changed since then and have not been sufficiently surveyed.

**Table 1:** The times of IRC publications under survey in prominent IRC network studies.

**Prominent IRC network studies**	**Authors**	**Times of IRC publications studied**
International collaboration in the sciences 1981–1985	Schubert and Braun, 1990	1981–1985
The measurement of international scientific collaboration	Luukkonen et al., 1993	1981–1986
Evolution of the social network of scientific collaborations	Barabâsi et al., 2002	1991–1998
The structure of scientific collaboration networks	Newman, 2001	1995–1999
Analyzing scientific networks through co-authorship	Glänzel and Schubert, 2004	1980, 1990, 2000
Network structure, self-organization, and the growth of international collaboration in science	Wagner and Leydesdorff, 2005	2000

### Studies About Research Collaborations Among CANZUK Countries

Previous studies have mentioned the research collaborations between CANZUK countries but no overall picture has emerged from all the studies. To get insights into IRC activities, data analytics have been essential in previous studies. Network maps have commonly been used to show the co-author relationships in bibliographic data. For example, AUS and NZL were grouped together as a separate cluster on global network maps of research (Davidson Frame and Carpenter, 1979) or in an “Anglo-saxon cluster” (GBR, CAN, IND, AUS, NZL and ZAF; Luukkonen et al., 1993). AUS and NZL also had a very strong research collaboration (Schubert and Braun, 1990; Luukkonen et al., 1993; Benckendorff, 2010). GBR had relatively more active RCs with AUS and NZL, and the IRC of GBR has been impacted more by historical connections than geographical proximity (Zitt et al., 2000). Conversely, GBR and AUS were in the top ten countries collaborating with Canada in nanotechnology during 1990–2009 (Hu et al., 2012).

The multidimensional scaling (MDS) technique has been used to investigate and describe the distances or dissimilarities between countries. As a two-dimensional map is often sufficient to describe the network of IRC relationships (Luukkonen et al., 1993), MDS is useful to visualize the possible effects of extra-scientific factors into an abstract Cartesian space. In detail, the MDS technique takes the “proximity” measures between countries regarding extra-scientific factors and produces a map in which “pairs of countries with a high volume of collaboration and a similar pattern of collaboration are placed close together, and dissimilar pairs with a low volume, far apart” (Davidson Frame and Carpenter, 1979). This technique shows close connections between CANZUK countries as the co-authorship map technique does. Although Australia and New Zealand have more active connections with countries in Western Europe and North America, these two countries have been grouped together as a separate cluster on a two-dimensional MDS map (Davidson Frame and Carpenter, 1979) or in an “Anglo-saxon cluster” (GBR, CAN, IND, AUS, NZL and ZAF; Luukkonen et al., 1993).

Although research connections of the above four countries have been mentioned separately in previous studies, little is known about whether the relationships among CANZUK countries differ from those between the CANZUK group with other countries. Consequently, it is unclear whether the CANZUK countries are stronger collaborators with each other than with other countries, just in the tourism field (Benckendorff, 2010) or in general as well.

### Measuring the Importance of Research Collaborations

As the relative *importance* of the collaborating countries for a given country can be characterized by the asymmetrical relationships among them (Glänzel and Schubert, 2001), the Affinity Index has been proposed to measure the relative interest between every pair of countries in international research collaboration (Okubo et al., 1992). The Affinity Index is calculated as follows:

A = C_xy_ / C_x_

where:

A : the Affinity Index, measuring the asymmetrical relationships between two countries, x and y.

C_xy_ : the observed number of research collaborations between a country (x) and its partners (y).

C_x_ : the total number of research collaborations carried out by country x.

By dividing pairwise collaborations by total collaborations, the above formula normalizes the number of research collaborations (RCs) between a country and its partner to arrive at values relative to the country's total numbers of collaborations. Reflecting the affinity toward partners, this measure highlights “the attractiveness of a partner in collaboration” (Zitt et al., 2000), or “the important partners in terms of quantity” (Chinchilla-Rodríguez et al., 2018). Therefore, this measure has been used to analyse the research partners of Asian countries (Arunachalam and Doss, 2000), or to examine the research collaborations between India and other countries in the field of Forensic Sciences (Jeyasekar and Saravanan, 2015).

### Counting the Research Collaborations Between Countries

Measuring the affinity index requires counting the observed number of research collaborations between every pair of countries, and the total number of research collaborations carried out by each country. Publications involving multiple authors with differing national backgrounds have been widely considered a conventional indicator to measure international research collaboration (Chen et al., 2019). However, co-authorship is just a partial indicator of collaboration (Katz and Martin, 1997) because collaboration does not necessarily lead to co-authored papers, and so co-authorship data does not fully reflect actual collaboration (Melin and Persson, 1996). Regarding the results of research, there are various types of outcomes beyond joint research publications: patents, joint research grants; (Yuan et al., 2018), and different rewards (Laudel, 2002) of the collaborations as contributionship: acknowledgments in PhD theses, research journals (articles, editorials, reviews, etc.) and books. However, as co-authored publications are considerably easier to analyze at scale, the present paper examines only such outputs when counting collaborations.

However, the countries involved in multinational publications could be credited differently by different methods. The difference in using these measurements, therefore, should be considered carefully in IRC studies.

There are two methods for counting authorship and thus collaboration among some countries, and thus for generating an affinity index: whole and fractional counting (Chinchilla-Rodríguez et al., 2021). The whole counting method credits one for every country participating in multinational publications. A publication co-authored by two researchers from Canada and three researchers from France can be taken as an example of this. At the country level, Canada is credited one and France is credited one using the whole counting method. On the other hand, the fractional counting method gets the credited values by dividing a multinational publication by the number of unique countries. In the example above, Canada is given one half of the credit for collaboration and France is credited one half for their involvement, using the fractional counting methods.

These two methods of counting have their own supporting arguments for use (Gauffriau, 2017). Therefore, there is no agreement for which methods should be used. Many studies calculating affinity index preferred the whole counting method while some other studies, assuming that the two methods give similar results, applied the fractional counting method (Chinchilla-Rodríguez et al., 2021).

## Materials and Methods

The purpose of the current study is to examine the changing importance of CANZUK‘s partners in research by answering three RQs mentioned above. This paper applies a quantitative approach using bibliographic data as follows:

### Data

We use Microsoft Academic Graph (MAG), which is among the most commonly used bibliographic data sources (Waltman and Larivière, 2020), to investigate the research collaborations of CANZUK countries. The reason (for choosing the MAG data set in this study) is because MAG is a general scholarly bibliographic data set that can be downloaded in whole from the Microsoft website, while the other data sources are behind the paywall. We discuss the limitations of using the MAG data set in the Conclusion Section below. We downloaded (in 2018) the entire MAG data set that was shared^2^ as part of Open Academic Graph v1. This data set comprises the bibliographic records of 166,192,182 publications with a total file size of 103 GB. Information about numbers of total publications and international co-authored publications were extracted from this data source and processed using the R statistical analysis program. To resolve the missing authors' country affiliation information in MAG, we used a method that enriches data by sourcing corresponding information from Wikidata (i.e., matches affiliation data like institution names to their parent countries), which we developed in previous work and validated with the MAG data set in particular (Nguyen et al., 2020). As most of the previous IRC network studies have examined the collaborative network maps in the period 1981–2000 (Table 1), this paper also compared the CANZUK's RCs in this period with those in the periods before and after. Therefore, the data from the years 1951 to 2017 was collected and separated into three time periods: 1951–1980, 1981–2000, and 2001–2017.

We then filtered the MAG data set to a subset of publications showing the international research collaboration related to the CANZUK countries in the above mentioned three periods. Tables 2A,B show the number and the percentage of publications in this subset, by period and by document type.

**Table 2A:** Summary of documents in the three periods (1951–1980, 1981–2000, 2001–2017) in the subset of IRC publications used for calculating the infinity index in this study.

**Period**	**No. of publications**	**% of publications**
1951–1980	16,323	1.21%
1981–2000	188,109	13.91%
2001–2017	1,147,599	84.88%
**Total**	**1,352,031**	**100.00%**

**Table 2B:** Summary of different document types in the subset of IRC publications used for calculating the infinity index in this study.

**Type**	**No. of publications**	**% of publications**
“Journal”	1,129,591	83.55%
“Conference”	77,888	5.76%
“BookReferenceEntry”	699	0.05%
“Book”	503	0.04%
Unknown (null)	143,350	10.60%
**Total**	**1,352,031**	**100.00%**

A summary of this partial data set, showing multi-author publications co-attributed to authors in each country, is also presented in Supplementary Table S2 (Appendix).

### Methods

There were three steps in this empirical study. In the first step, we generated the network maps of international co-authorship relationships relating to CANZUK countries. We used two methods for quantifying RCs: whole counting and fractional counting. The network analysis technique was applied to create the maps of research networks over the three different periods mentioned above.

In the second step, we calculated and compared the asymmetric relationships of partners for each CANZUK country in the three corresponding periods. The Affinity Index (described above) was applied to measure these research collaborations.

In the third step we measured and compared (1) the asymmetric relationships among the CANZUK countries only to (2) the relationships between other countries and the CANZUK group.

## Results

### Overview of CANZUK Collaboration Network Map

**Figure 1:**
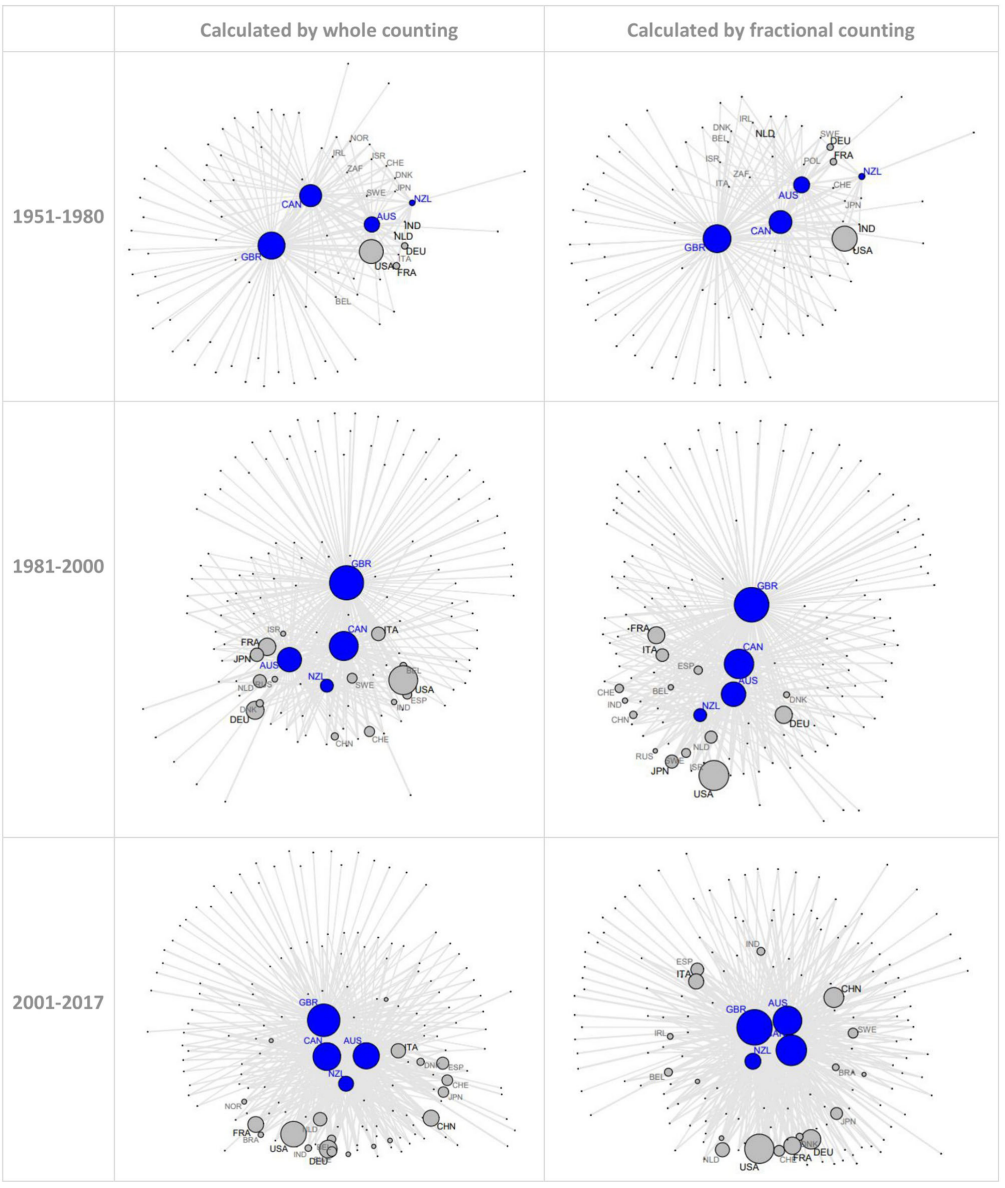
Network maps of international co-authorship relationships relating to CANZUK countries in three periods: 1951–1980, 1981–2000, and 2001–2017 (top 15 collaborative countries and the CANZUK countries were labeled: the top 5 in black and the next 10 in gray. CANZUK countries were labeled in blue). The number of research connections has increased over time.

Figure 1 shows the maps of research collaborations involving CANZUK countries (i.e., at least one country in the research connections is a CANZUK country) over time. Each country is represented as a node, and the research relationship between two countries is represented as an edge connecting the corresponding two nodes. The node size reflects the logarithm of the ratio of the corresponding countries' IRC numbers to their median value in the same map (i.e., the countries' relative volume of international research collaborations, and thus relative IRC frequency, with all partner countries). The thickness of the edges reflects the logarithm of ratios of IRC numbers from pairs of countries to their median value (i.e., the relative IRC frequency between two countries). To simplify the visualization, only the CANZUK countries and the top 15 countries having the highest number of total research collaborations were labeled on the maps. Also, only the edges having values greater than the median values were presented on the maps. The maps calculated by either whole counting method or fractional method show similar results. The results show that the number of research connections has increased over time, and that the USA was notably the most collaborative country in all three periods. GBR and CAN were also two important collaborative partners among CANZUK countries. Meanwhile, AUS and NZL ranked third and fourth respectively regarding their numbers of internationally collaborative publications.

### The Asymmetric Relationships of Partners Among CANZUK Countries

**Figure 2:**
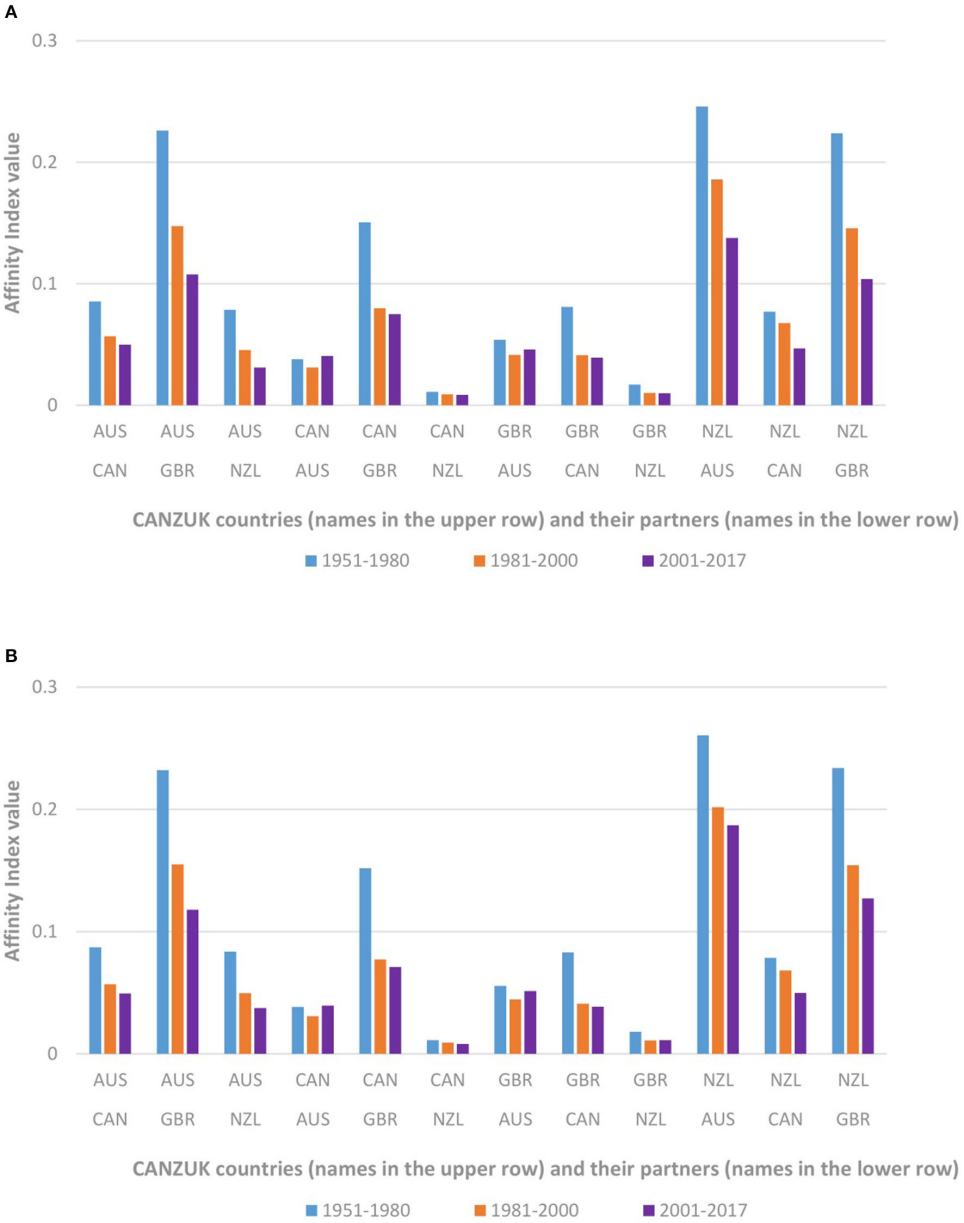
The affinity index values of CANZUK countries, calculated by **(A)** whole counting, and **(B)** fractional counting. The affinity index values of research collaborations between CANZUK countries and their counterparts were decreasing over time.

Figures 2A,B shows the Affinity Index of collaborative countries among the CANZUK in the three periods: 1951–1980, 1981–2000, and 2001–2017, calculated by whole counting and fractional counting respectively. The clustered column charts display a series of affinity indexes, corresponding to the values at the three periods, for each asymmetric relationship. The higher the columns are, the more important the partners are to the CANZUK countries.

In the first period, AUS and GBR were notably important to NZL, while GBR was also an important collaborator of AUS (the corresponding affinity index values were higher than 0.2). In the second and third periods, these partners were still important (all corresponding affinity index values were higher than 0.1, in comparison to those of other CANZUK countries' research collaborations).

The detailed values of affinity index values of CANZUK countries calculated by the two counting methods were presented in Supplementary Tables S3, S4 (Appendix).

Both counting methods described similar trends. In general, the affinity index values (of research collaborations between CANZUK countries and their counterparts) were decreasing over time in this figure. However, the interpretation that CANZUK countries have been less common partners to their counterparts in the group might be incorrect. The reason is that, as each country has developed research collaboration with more other countries over time, the average proportion in research collaboration between that country and its partners has declined. Consequently, comparing the affinity index values over periods is not appropriate. Therefore, a further normalization across periods is needed, and is represented in the following section.

### Examining the Development of CANZUK Partners' Asymmetric Relationships Over Time

To examine how the importance of CANZUK countries' partners has changed across the three periods mentioned, this paper compared the CANZUK affinity index values to the total values. In detail, this paper extracted information about the two groups of research collaborations. The first group includes the research collaborations among CANZUK countries. The second group includes the research collaborations between CANZUK countries and other countries. The total of these two groups included all research collaborations in which at least one CANZUK country was involved in the research connections, as presented in Figure 1.

Two types of values were calculated, and were compared against the total values. They were medians and standard deviations of affinity index calculated for the above two groups of research collaborations. As explained above, these values needed to be normalized for comparison across periods by calculating the ratios of each group's medians and standard deviations to the corresponding values of the total two groups. While the ratios of medians could show whether the importance of one research collaboration group has been increased, the ratios of standard deviations could inform how much the affinity index values spread out from the trend line. Tables 3, 4 show the ratios using the whole counting method and fractional counting method, respectively.

**Table 3:** Comparing the ratios of medians and standard deviations of affinity index values of each group to the values of the total all countries, using the whole counting method.

		**1951–1980**	**1981–2000**	**2001–2017**
Among CANZUK countries	Ratio of Group 1's affinity index median to the total all countries' median	68.42	172.59	237.62
(Group 1)	Ratio of Group 1's standard deviation to the total all countries' standard deviation	1.95	2.22	2.38
CANZUK countries with other	Ratio of Group 2's affinity index median to the total all countries' median	1.00	0.98	0.95
countries (Group 2)	Ratio of Group 2's standard deviation to the total all countries' standard deviation	0.86	0.90	0.90

*The trends expressed by the ratio of affinity index medians to the total all countries' medians are not clear*.

**Table 4:** Comparing the ratios of medians and standard deviations of affinity index values of each group to the values of the total all countries, using the fractional counting method.

		**1951–1980**	**1981–2000**	**2001–2017**
Among CANZUK countries	Ratio of Group 1's affinity index median to the total all countries' median	66.00	197.60	337.08
(Group 1)	Ratio of Group 1's standard deviation to the total all countries' standard deviation	1.97	2.24	2.69
CANZUK countries with other	Ratio of Group 2's affinity index median to the total all countries' median	0.96	0.94	0.96
countries (Group 2)	Ratio of Group 2's standard deviation to the total all countries' standard deviation	0.86	0.90	0.89

*There is an increasing trend for the importance of CANZUK countries to their counterparts in the group, and a decreasing trend for the importance of other countries to the CANZUK group*.

The two tables suggest that the importance of CANZUK partners to CANZUK countries has rapidly increased over time. In both tables, the median ratios show a clearly increasing trend for the importance of CANZUK countries to their counterparts in the group, and a decreasing trend for the importance of other countries to the CANZUK group. However, the values calculated by the fractional counting method show a little noise in the period 2001–2017 when the importance of other countries to the CANZUK group saw a small increase (Table 4). The differences between the values calculated by the two counting methods suggest that in the last period, the proportion of publications credited to the other countries collaborating with the CANZUK was larger than their proportion of international research relationships.

The series values of standard deviation ratios of Group 1's ratio have slightly increased over time while those of Group 2's ratio tend to be unchanged. In other words, there was a tendency that the importance of CANZUK countries to their counterparts gradually spread out over time.

## Discussion

The goal of the present study is to examine the importance of research partners to CANZUK countries. The main findings are discussed here with regards to the research question they answer.

RQ1: How has the importance of partners for research collaborations among CANZUK countries developed over time?

The present study found that GBR has always been the most important collaborative partner to other countries across the three surveyed periods. One exception is the case of NZL, to which AUS has been the most important collaborative partner. The study's results also illustrated that the importance of partners in research collaborations among CANZUK countries has increased over time. This finding has not been mentioned in previous studies. The increasing importance among CANZUK countries is consistent with the previous finding that the CANZUK countries have been likely impacted by historical connections (Zitt et al., 2000). The research collaborations between CANZUK countries are even expected to develop more in the future (Bell and Vucetic, 2019), especially since GBR has left the EU and is looking for new potential research collaborators (Garas et al., 2019).

RQ2: How has the importance of partners for research collaborations between CANZUK and other countries developed over time?

For each country, the importance of its partners' research collaborations should be evaluated so that the policy makers could decide future research policy. As a result of the increasing importance of partners in research collaborations among CANZUK countries (Group 1), the importance of other countries to CANZUK countries (Group 2) has decreased over time, as shown in Table 3. In this zero-sum game, the CANZUK countries have recently strengthened the relationships with their traditional allies at the cost of disregarding the other partners (Perot, 2021). However, the pace of Group 1's increased gaps (i.e., the speed of gaps' changes) over the three periods is much larger in comparison to that of Group 2's decreased gaps. In Table 3, the ratio of Group 1's affinity index median to the total all countries' median has increased at 68.42, 172.59, and 237.62 in the periods 1951–1980, 1981–2000, and 2001–2017 respectively. Meanwhile, the ratio of Group 2's has decreased with the corresponding values 1.00, 0.98, and 0.95. In other words, while the “middle” value of Group 1's affinity indexes has quickly become higher, the “middle” value of Group 2's affinity indexes has slightly become lower, than the “middle value” of affinity indexes of all countries. The difference in the pace of the above mentioned gaps can be explained by the higher number of countries in Group 2 in comparison with only four CANZUK countries in Group 1, so the middle value of Group 2's affinity indexes is very close to the middle value of all counties' affinity indexes. Although the CANZUK countries have strengthened the research relationships with their CANZUK partners, the decrease in their research relationships with other countries will be so slight as to be unnoticeable. The implication here is that, as CANZUK countries have moved to strengthen their Anglo-American strategic alliance, other countries should pay attention to any changes in their relations with CANZUK countries.

RQ3: How do different methods of counting research collaborations show different results?

The two methods of counting research collaborations resulted in similar outcomes. However, there were some slight differences in the results. For example, the median ratios calculated by the whole counting method show an increasing trend for the importance of CANZUK countries to their counterparts in the group, and a decreasing trend for the importance of other countries to the CANZUK group. Meanwhile, the values calculated by the fractional counting method show less clear trends, with little noise at the last period for the importance of other countries to the CANZUK group. These differences could be explained by the nature of the two different methods of counting. During the period 2001–2017, the increase of collaborative links (credited to countries by whole counting) between CANZUK countries and other countries might be less than the increase of the total collaborative links. Therefore, the ratio of Group 2's affinity index median to the total all countries' median was slightly reduced from 0.98 to 0.95. Nonetheless, the increase of co-authored publications (credited to countries by fractional counting) between CANZUK countries and other countries might be still more than the increase of the total co-authored publications. This increase was reflected with the corresponding ratio value from 0.94 to 0.96 in the last period. A possible recommendation here is that the whole counting method may be best applied for relationship-based measurement (e.g., evaluating the research relationships between countries) while the fractional counting method may be best applied for production-based measurement (e.g., comparing the publications between countries).

Counting methods are the underlying methods that need to be implemented in any IRC measurements to analyse the research collaboration patterns across countries (e.g., association strength, inclusion index, Jaccard index, Salton index), so the use of a particular counting method might change the results that could be obtained by the other method. Although the differences given by the two counting methods in this study were not notable, the cautiousness in choosing a suitable counting method may be necessary in other IRC studies. For example, further research about how different IRC measurements are used to analyse the RC patterns across countries could also compare the effects of choosing counting methods in their implementations.

## Conclusion

In conclusion, the present study investigated how the importance of CANZUK countries' partners has changed over time by analyzing them using both the whole counting method and the fractional counting method. The study revealed that the CANZUK countries have been more important partners to their counterparts within the group, while other countries have been less important partners to the CANZUK group. Another finding was that the two methods of counting the research collaborations between countries resulted in slightly different outcomes. Although these differences do not affect the interpretation about the overall increase of CANZUK partners' importance to CANZUK countries in this study, there is no promise that the two counting methods will still give similar conclusions in other research. Therefore, carefully choosing a suitable counting method may be necessary for other IRC studies, especially if they focus on relationship-based measurement (i.e., choosing the whole counting method) or production-based measurement (i.e., choosing the fractional counting method).

There are limitations in the present study as well as opportunities for further research. First, this study chose the MAG data source to examine the importance of the CANZUK countries' research collaborations. Consequently, the results from this choice may be different than those obtained if other data sources were used. A study about the effects of data set choice on measuring IRC has shown that different data sources give slightly different outcomes of IRC measurement (Nguyen et al., 2019). Second, this study examined countries other than the CANZUK countries as a single group, and concluded that the importance of this group to the CANZUK countries has decreased over time. Therefore, exploring the importance of each CANZUK country's partner in this group, or at least the top collaborative countries, should be a focus of future studies. Despite these limitations, the present study is important because it provides new insights into the importance of collaborative partners to CANZUK countries: the study revealed that the CANZUK countries, in comparison with the other countries, have been more important partners to their counterparts in the group. In other words, the group of CANZUK countries is becoming more of a “closed shop” than a “collaborative hub” (i.e., collaborating within the group more than promoting collaborations with countries outside the group).

While identifying and analyzing the important partners of CANZUK countries was the focus of this study, a further relevant research area is the relative strengths of countries that have disproportionately strong connections (Chinchilla-Rodríguez et al., 2018) with CANZUK countries. Different similarity measures normalized by the total numbers of research collaborations carried out by both connected countries (e.g., association strength, inclusion index, Jaccard index, and cosine index) can be used to examine the relative strengths of countries' collaborations within this particular research area. Given that the sizes of both countries are included in calculating the relative strength, such measures are not size-dependent. Therefore, further studies in this relevant research area can complement the knowledge from the present study by revealing the drivers underlying the formation of collaborations (e.g., cultural proximity or linguistic proximity) between every two countries under survey (Chinchilla-Rodríguez et al., 2018).

## Data Availability Statement

The datasets presented in this study can be found in online repositories. The names of the repository/repositories and accession number(s) can be found below: https://github.com/baxuan/IRC-Importance-Measurement.

## Author Contributions

BN, JD, and ML-R contributed to conception of the study. BN designed the study, organised the research data, performed the statistical analyses, and wrote the first draft of the manuscript. All authors contributed to manuscript revision, read, and approved the submitted version.

## Conflict of Interest

The authors declare that the research was conducted in the absence of any commercial or financial relationships that could be construed as a potential conflict of interest.

## Publisher's Note

All claims expressed in this article are solely those of the authors and do not necessarily represent those of their affiliated organizations, or those of the publisher, the editors and the reviewers. Any product that may be evaluated in this article, or claim that may be made by its manufacturer, is not guaranteed or endorsed by the publisher.

## Footnotes

^1^The countries in this study will be referred to by their corresponding three-letter country abbreviations, as listed in Supplementary Table S1 (Appendix).

^2^ https://www.aminer.org/open-academic-graph

## References

[B1] Arunachalam S. Doss M. J. (2000). Mapping international collaboration in science in Asia through coauthorship analysis. Curr. Sci. 79, 621–628. Available online at: http://www.jstor.org/stable/24105078

[B2] Barabâsi A. L. Jeong H. Néda Z. Ravasz E. Schubert A. Vicsek T. (2002). Evolution of the social network of scientific collaborations. Physica A: Statist. Mech. Appl. 311, 590–614. 10.1016/S0378-4371(02)00736-7

[B3] Baty C. K. Lane M. Cater-Steel A. Ally M. (2017). The role of national culture in the strategic use of and investment in ICT: a comparative study of Japanese and Australian organisations, in Proceedings of the 28th Australasian Conference on Information Systems (ACIS 2017), Australian Association for Information Systems.

[B4] Bell D. Vucetic S. (2019). Brexit, CANZUK, and the legacy of empire. Br. J. Polit. Int. Relat. 21, 367–382. 10.1177/1369148118819070

[B5] Benckendorff P. (2010). Exploring the limits of tourism research collaboration: a social network analysis of co-authorship patterns in Australian and New Zealand tourism research, in Tourism and hospitality: Challenge the Limits Conference, Tasmania, Australia, 8–11.

[B6] Castillo J. A. Powell M. A. (2020). Research impact and international collaboration: a study of ecuadorian science. J. Hisp. Higher Educ. 19, 232–249. 10.1177/1538192718779169

[B7] Chen K. Zhang Y. Fu X. (2019). International research collaboration: an emerging domain of innovation studies?. Res. Policy 48, 149–168. 10.1016/j.respol.2018.08.00532723851

[B8] Chinchilla-Rodríguez Z. Bu Y. Robinson-García N. Costas R. Sugimoto C. R. (2018). Travel bans and scientific mobility: utility of asymmetry and affinity indexes to inform science policy. Scientometrics 116, 569–590. 10.1007/s11192-018-2738-2

[B9] Chinchilla-Rodríguez Z. Bu Y. Robinson-García N. Sugimoto C. R. (2021). An empirical review of the different variants of the probabilistic affinity index as applied to scientific collaboration. Scientometrics 126, 1775–1795. 10.1007/s11192-020-03815-9

[B10] Davidson Frame J. Carpenter M. P. (1979). International research collaboration. Social Stud. Sci. 9, 481–497. 10.1177/030631277900900405

[B11] Garas G. Cingolani I. Patel V. M. Panzarasa P. Darzi A. Athanasiou T. (2019). Evaluating the implications of Brexit for research collaboration and policy: a network analysis and simulation study. BMJ Open 9, e025025. 10.1136/bmjopen-2018-025025 31506256 PMC6747879

[B12] Gauffriau M. (2017). A categorization of arguments for counting methods for publication and citation indicators. J. Inform. 11, 672–684. 10.1016/j.joi.2017.05.009

[B13] Glänzel W. Schubert A. (2001). Double effort = double impact? A critical view at international co-authorship in chemistry. Scientometrics 50, 199–214. 10.1023/A:1010561321723

[B14] Glänzel W. Schubert A. (2004). Analysing scientific networks through co-authorship, in Handbook of Quantitative Science and Technology Research (Dordrecht: Springer), 257–276. 10.1007/1-4020-2755-9_12

[B15] Hatakenaka S. (2008). New developments in international research collaboration. Int. Higher Educ. 50. 10.6017/ihe.2008.50.7998

[B16] Hu G. Carley S. Tang L. (2012). Visualizing nanotechnology research in Canada: evidence from publication activities (1990–2009). J. Technol. Transfer 37, 550–562. 10.1007/s10961-011-9238-3

[B17] Jeyasekar J. J. Saravanan P. (2015). Impact of collaboration on indian forensic science research: a scientometric mapping from 1975 to 2012. J. Sci. Res. 4, 135–142. 10.4103/2320-0057.174863

[B18] Katz J. S. Martin B. R. (1997). What is research collaboration? Res. Policy 26, 1–18. 10.1016/S0048-7333(96)00917-1

[B19] Laudel G. (2002). What do we measure by co-authorships?. Res. Eval. 11, 3–15. 10.3152/147154402781776961

[B20] Luukkonen T. Tijssen R. Persson O. Sivertsen G. (1993). The measurement of international scientific collaboration. Scientometrics 28, 15–36. 10.1007/BF02016282

[B21] Melin G. Persson O. (1996). Studying research collaboration using co-authorships. Scientometrics 36, 363–377. 10.1007/BF0212960030912999

[B22] Newman M. E. (2001). The structure of scientific collaboration networks. PNAS. 98, 404–409. 10.1073/pnas.98.2.40 11149952 PMC14598

[B23] Nguyen B. X. Dinneen J. D. Luczak-Roesch M. (2020). A novel method for resolving and completing authors' country affiliation data in bibliographic records. J. Data Inform. Sci. 5, 97–115. 10.2478/jdis-2020-0020

[B24] Nguyen B. X. Luczak-Roesch M. Dinneen J. D. (2019). Exploring the effects of data set choice on measuring international research collaboration: An example using the ACM digital library and microsoft academic graph. arXiv [Preprint]. arXiv: 1905.12834. Available online at: https://arxiv.org/ftp/arxiv/papers/1905/1905.12834.pdf

[B25] Okubo Y. Miquel J. Frigoletto L. Doré J. (1992). Structure of international collaboration in science: typology of countries through multivariate techniques using a link indicator. Scientometrics 25, 321–351. 10.1007/BF02028090

[B26] Perot E. (2021). The Aukus Agreement, What Repercussions for the European Union. The Robert Schuman Foundation-European Issue. Brussels: Foundation Robert Schuman.

[B27] Pohl H. (2020). Collaboration with countries with rapidly growing research: supporting proactive development of international research collaboration. Scientometrics 122, 287–307. 10.1007/s11192-019-03287-6

[B28] Schubert A. Braun T. (1990). International collaboration in the sciences 1981–1985. Scientometrics 19, 3–10. 10.1007/BF02130461

[B29] Wagner C. S. Leydesdorff L. (2005). Mapping the network of global science: comparing international co-authorships from 1990 to 2000. Int. J. Technol. Global. 1, 185–208. 10.1504/IJTG.2005.007050

[B30] Waltman L. Larivière V. (2020). Special issue on bibliographic data sources. Quant. Sci. Stud. 1, 360–362. 10.1162/qss_e_00026

[B31] Yuan L. Hao Y. Li M. Bao C. Li J. Wu D. (2018). Who are the international research collaboration partners for China? A novel data perspective based on NSFC grants. Scientometrics 116, 401–422. 10.1007/s11192-018-2753-3

[B32] Zitt M. Bassecoulard E. Okubo Y. (2000). Shadows of the past in international cooperation: Collaboration profiles of the top five producers of science. Scientometrics 47, 627–657. 10.1023/A:1005632319799

