# Supplementary material for: Closed Shop or Collaborative Hub? An Analysis of the Partners' Importance in CANZUK Countries' Research Collaborations
Source: Front Res Metr Anal. 2022 Jul 26;7:838553. doi: 10.3389/frma.2022.838553 (PMC9360419; doi:10.3389/frma.2022.838553)
Supplement: Supplementary file 1 [file Table_1.pdf]

Supplementary Table S1. List of the current officially assigned ISO 3166-1 alpha-3 codes of the CANZUK countries and their corresponding English country names.

ISO 3166-1 alpha-3 codes	English country names
AUS	Australia, officially the Commonwealth of Australia
CAN	Canada
GBR	The United Kingdom of Great Britain and Northern Ireland, commonly known as the United Kingdom (UK) or Britain
NZL	New Zealand
