# Supplementary material for: Closed Shop or Collaborative Hub? An Analysis of the Partners' Importance in CANZUK Countries' Research Collaborations
Source: Front Res Metr Anal. 2022 Jul 26;7:838553. doi: 10.3389/frma.2022.838553 (PMC9360419; doi:10.3389/frma.2022.838553)
Supplement: Supplementary file 2 [file Table_2.pdf]

Supplementary Table S2. Summary of publications (number of publications and percentage) per country in the subset of IRC publications used for calculating the infinity index in this study. Number of publications indicates the publications that a country has authors involved in producing them. The countries are presented by their ISO 3166-1 alpha-3 codes.

Country	Number of publications	Percentage
ABW	8	0.00%
AFG	114	0.01%
AGO	86	0.01%
AIA	9	0.00%
ALA	1	0.00%
ALB	159	0.01%
AND	17	0.00%
ARE	4,000	0.30%
ARG	7,473	0.55%
ARM	1,513	0.11%
ASM	11	0.00%
ATA	6	0.00%
ATF	1	0.00%
ATG	16	0.00%
AUS	306,289	22.65%
AUT	24,019	1.78%
AZE	629	0.05%
BDI	37	0.00%
BEL	39,974	2.96%
BEN	285	0.02%
BES	0	0.00%
BFA	671	0.05%
BGD	3,255	0.24%
BGR	3,195	0.24%

Country	Number of publications	Percentage
LBN	1,831	0.14%
LBR	66	0.00%
LBY	514	0.04%
LCA	201	0.01%
LIE	69	0.01%
LKA	2,497	0.18%
LSO	40	0.00%
LTU	1,980	0.15%
LUX	1,383	0.10%
LVA	776	0.06%
MAC	313	0.02%
MAF	0	0.00%
MAR	1,457	0.11%
MCO	202	0.01%
MDA	201	0.01%
MDG	374	0.03%
MDV	38	0.00%
MEX	10,577	0.78%
MHL	79	0.01%
MKD	431	0.03%
MLI	346	0.03%
MLT	945	0.07%
MMR	227	0.02%
MNE	186	0.01%

BHR	361	0.03%
BHS	98	0.01%
BIH	274	0.02%
BLM	0	0.00%
BLR	1,322	0.10%
BLZ	63	0.00%
BMU	136	0.01%
BOL	388	0.03%
BRA	27,264	2.02%
BRB	294	0.02%
BRN	367	0.03%
BTN	133	0.01%
BVT	0	0.00%
BWA	505	0.04%
CAF	40	0.00%
CAN	407,114	30.11%
CCK	2	0.00%
CHE	56,110	4.15%
CHL	10,129	0.75%
CHN	117,500	8.69%
CIV	298	0.02%
CMR	949	0.07%
COD	316	0.02%
COG	175	0.01%
COK	487	0.04%
COL	3,890	0.29%
COM	6	0.00%
CPV	37	0.00%

MNG	388	0.03%
MNP	6	0.00%
MOZ	362	0.03%
MRT	22	0.00%
MSR	31	0.00%
MTQ	31	0.00%
MUS	343	0.03%
MWI	1,422	0.11%
MYS	12,867	0.95%
MYT	0	0.00%
NAM	438	0.03%
NCL	387	0.03%
NER	175	0.01%
NFK	331	0.02%
NGA	3,406	0.25%
NIC	122	0.01%
NIU	8	0.00%
NLD	80,747	5.97%
NOR	24,525	1.81%
NPL	1,147	0.08%
NRU	13	0.00%
NZL	63,009	4.66%
OMN	1,297	0.10%
PAK	6,094	0.45%
PAN	542	0.04%
PCN	0	0.00%
PER	2,327	0.17%
PHL	2,320	0.17%

CRI	731	0.05%
CUB	935	0.07%
CUW	8	0.00%
CXR	1	0.00%
CYM	29	0.00%
CYP	2,831	0.21%
CZE	13,260	0.98%
DEU	151,516	11.21%
DJI	20	0.00%
DMA	27	0.00%
DNK	35,120	2.60%
DOM	124	0.01%
DZA	1,234	0.09%
ECU	805	0.06%
EGY	6,015	0.44%
ERI	48	0.00%
ESH	1	0.00%
ESP	66,401	4.91%
EST	2,822	0.21%
ETH	1,466	0.11%
FIN	22,622	1.67%
FJI	808	0.06%
FLK	83	0.01%
FRA	118,554	8.77%
FRO	128	0.01%
FSM	31	0.00%
GAB	197	0.01%
GBR	735,350	54.39%

PLW	40	0.00%
PNG	1,003	0.07%
POL	21,495	1.59%
PRI	804	0.06%
PRK	411	0.03%
PRT	17,019	1.26%
PRY	113	0.01%
PSE	348	0.03%
PYF	162	0.01%
QAT	2,727	0.20%
REU	56	0.00%
ROU	5,014	0.37%
RUS	23,497	1.74%
RWA	288	0.02%
SAU	9,037	0.67%
SDN	583	0.04%
SEN	397	0.03%
SGP	18,960	1.40%
SGS	17	0.00%
SHN	8	0.00%
SJM	12	0.00%
SLB	205	0.02%
SLE	245	0.02%
SLV	91	0.01%
SMR	9	0.00%
SOM	17	0.00%
SPM	0	0.00%
SRB	2,908	0.22%

GEO	4,020	0.30%	SSD	39	0.00%
GGY	24	0.00%	STP	10	0.00%
GHA	2,208	0.16%	SUR	35	0.00%
GIB	46	0.00%	SVK	3,837	0.28%
GIN	98	0.01%	SVN	4,235	0.31%
GLP	34	0.00%	SWE	48,917	3.62%
GMB	963	0.07%	SWZ	105	0.01%
GNB	81	0.01%	SXM	11	0.00%
GNQ	29	0.00%	SYC	141	0.01%
GRC	21,454	1.59%	SYR	475	0.04%
GRD	63	0.00%	TCA	16	0.00%
GRL	195	0.01%	TCD	50	0.00%
GTM	242	0.02%	TGO	77	0.01%
GUF	45	0.00%	THA	9,542	0.71%
GUM	61	0.00%	TJK	34	0.00%
GUY	76	0.01%	TKL	1	0.00%
HKG	12,237	0.91%	TKM	7	0.00%
HMD	0	0.00%	TLS	103	0.01%
HND	91	0.01%	TON	66	0.00%
HRV	3,359	0.25%	TTO	525	0.04%
HTI	57	0.00%	TUN	1,545	0.11%
HUN	10,929	0.81%	TUR	11,645	0.86%
IDN	3,984	0.29%	TUV	35	0.00%
IMN	56	0.00%	TWN	12,953	0.96%
IND	32,997	2.44%	TZA	2,483	0.18%
IOT	0	0.00%	UGA	2,490	0.18%
IRL	24,925	1.84%	UKR	4,180	0.31%
IRN	14,855	1.10%	UMI	4	0.00%

IRQ	991	0.07%
ISL	2,544	0.19%
ISR	18,776	1.39%
ITA	91,688	6.78%
JAM	591	0.04%
JEY	2,870	0.21%
JOR	1,991	0.15%
JPN	58,979	4.36%
KAZ	606	0.04%
KEN	4,891	0.36%
KGZ	87	0.01%
KHM	604	0.04%
KIR	32	0.00%
KNA	38	0.00%
KOR	20,172	1.49%
KWT	1,549	0.11%
LAO	402	0.03%

URY	953	0.07%
USA	464,570	34.36%
UZB	294	0.02%
VAT	20	0.00%
VCT	617	0.05%
VEN	1,322	0.10%
VGB	35	0.00%
VIR	6	0.00%
VNM	3,184	0.24%
VUT	119	0.01%
WLF	6	0.00%
WSM	98	0.01%
YEM	142	0.01%
ZAF	20,027	1.48%
ZMB	774	0.06%
ZWE	1,158	0.09%
Total	1,352,031	100%
