# Supplementary material for: Closed Shop or Collaborative Hub? An Analysis of the Partners' Importance in CANZUK Countries' Research Collaborations
Source: Front Res Metr Anal. 2022 Jul 26;7:838553. doi: 10.3389/frma.2022.838553 (PMC9360419; doi:10.3389/frma.2022.838553)
Supplement: Supplementary file 3 [file Table_3.pdf]

Supplementary Table S3. Affinity Index of CANZUK countries in the three periods (1951-1980, 1981-2000, 2001-2017), calculated by whole counting.

Country	Partner	1951-1980	1981-2000	2001-2017
AUS	CAN	0.085	0.057	0.050
AUS	GBR	0.226	0.147	0.108
AUS	NZL	0.079	0.045	0.031
CAN	AUS	0.038	0.031	0.040
CAN	GBR	0.151	0.080	0.075
CAN	NZL	0.011	0.009	0.009
GBR	AUS	0.054	0.041	0.046
GBR	CAN	0.081	0.041	0.039
GBR	NZL	0.017	0.010	0.010
NZL	AUS	0.246	0.186	0.138
NZL	CAN	0.077	0.068	0.047
NZL	GBR	0.224	0.146	0.104
