# Supplementary material for: Closed Shop or Collaborative Hub? An Analysis of the Partners' Importance in CANZUK Countries' Research Collaborations
Source: Front Res Metr Anal. 2022 Jul 26;7:838553. doi: 10.3389/frma.2022.838553 (PMC9360419; doi:10.3389/frma.2022.838553)
Supplement: Supplementary file 4 [file Table_4.pdf]

Supplementary Table S4. Affinity Index of CANZUK countries in the three periods (1951-1980, 1981-2000, 2001-2017), calculated by fractional counting.

Country	Partner	1951-1980	1981-2000	2001-2017
AUS	CAN	0.087	0.057	0.049
AUS	GBR	0.232	0.155	0.118
AUS	NZL	0.084	0.050	0.038
CAN	AUS	0.038	0.031	0.040
CAN	GBR	0.152	0.077	0.071
CAN	NZL	0.011	0.009	0.008
GBR	AUS	0.056	0.045	0.051
GBR	CAN	0.083	0.041	0.039
GBR	NZL	0.018	0.011	0.011
NZL	AUS	0.260	0.202	0.187
NZL	CAN	0.079	0.068	0.050
NZL	GBR	0.234	0.154	0.127
